# Are Many Hospitals to Be Closed?

**Published:** 1919-06-28

**Authors:** George Burford

**Affiliations:** 35 Queen Anne Street, W. 1.


*To the Editor of* THE HOSPITAL.

SIR,—In your very interesting and important article, "Professional Secrecy," you state that any syphilitic person who knowingly marries commits an infamous act.

What of the priest who performs the rite, knowing that, say, the man is infected with syphilis?

I know a priest who did this although I warned him.

Yours,

Swansea. E. ASHTON STEPHENS.

### "Are Many Hospitals to be Closed?"

*To the Editor of* THE HOSPITAL.

SIR,—Your courteous animadversion under this head in your issue of June 14 on a portion of my remarks at the annual meeting of the Bristol Homœopathic Hospital was *prima facie* justifiable; the paradox of an increased hospital service with a lessened aggregate of institutions was too patent to escape criticism. But this is no solitary instance wherein the disseverance of text from context, due to stress of reporting, while conducing to brevity, has obscured the argument.

Among the lessons of the recent influenzal and other epidemics are plainly and demonstrably how dreadful has been the inadequacy of hospital provision for acute and dangerous illness, and how the spread of infection and the avoidable mortality has been thus greatly augmented! The statesmanlike course is for the Ministry of Health to concern itself with the effective provision of surgical, medical, and nursing requirement for every man, woman, and child in Great Britain. This is *salus populi suprema lex* on the grand scale—adequacy to which this country has been totally unaccustomed.

Unification making for adequacy such as this inevitably requires centralised administration also on the grand scale; and centralised provision so vast inevitably necessitates the scrapping of many of the smaller voluntary hospital units which have been outposts in the fight against disease and death. The multiplicity of such separate hospitals, each with its separate organisation and equipment; the numerous and various hospital staffs; the competitive appeals to subscribers; all these involve much wasteful overlapping in civic hospital life, which centralised administration and institutions should obviate.

How readily a great State medical service might incidentally be inimical to spontaneity in medical development is seen in paragraph 23 of the scheme for a Ministry of Health issued by the British Medical Association:

"That no hospital shall in future be established in any area unless cause has been shown to the satisfaction of the Ministry of Health that a hospital is desirable in such area, and then only after a thorough investigation by the Advisory Council."

From such a suggestion I emphatically dissent; and though its adoption would doubtless ease the work of national administration, it would be at the cost of that freedom for development which is the basis of scientific progress.

Believe me,

Yours very faithfully,

GEORGE BURFORD, M.B.

35 Queen Anne Street, W. 1.

